# Predicting Hospital Outcomes Using the Reported Edmonton Frail Scale-Thai Version

**DOI:** 10.1093/geroni/igaa057.963

**Published:** 2020-12-16

**Authors:** Inthira Roopsawang, Hilaire Thompson, Oleg Zaslavsky, Basia Belza, Suparb Aree-Ue

**Affiliations:** 1 Mahidol University, Bangkok, Thailand; 2 University of Washington, Seattle, Seattle, Washington, United States; 3 University of Washington, Seattle, Washington, United States; 4 Mahidol, Bangkok, Thailand

## Abstract

Backgrounds Frailty is a common geriatric condition leading to poor surgical outcomes. Having a valid frailty measure has the potential to improve surgical care quality. Objectives To test the ability of the Reported Edmonton Frailty Scale-Thai version (REFS-Thai) in predicting hospital outcomes compared with the American Society of Anesthesiologists physical status classification (ASA) and the Elixhauser Comorbidity Measure (EMC) in older Thai orthopedic patients. Methods A prospective study was conducted on hospitalized older adults scheduled for elective orthopedic surgery. Multiple Firth logistic regression modeled the effect of frailty on postoperative complications, postoperative delirium (POD), and discharge disposition, while the length of stay (LOS) was examined by Poisson regression. The area under the receiver operating characteristic curve (AUC) and mean squared errors (MSE) were used to compare the predictive ability of the instruments. Results Two hundred participants with mean age of 72 (range 60-94 years) were mostly female, 23% were frail. Adjusting for other variables, frailty was significantly associated with postoperative complications (OR = 2.38, p = 0.049), POD (OR = 3.52, p = 0.034), and prolonged LOS (relative risk [RR] = 1.42, p = 0.043). The REFS-Thai alone shows good performance in predicting postoperative complications (AUC = 0.81, 95% CI = 0.74-0.88) and POD (AUC = 0.81, 95% CI = 0.72-0.90). The combination of REFS-Thai with ASA and EMC demonstrates an improved predictability. Conclusion The REFS-Thai was useful in predicting adverse outcomes in surgical orthopaedic older adults. Integrating the REFS-Thai for preoperative assessment may be useful for enhancing care quality.

